# The Polemic Diagnostic Role of *TP53* Mutations in Liquid Biopsies from Breast, Colon and Lung Cancers

**DOI:** 10.3390/cancers12113343

**Published:** 2020-11-12

**Authors:** M. Carmen Garrido-Navas, Abel García-Díaz, Maria Pilar Molina-Vallejo, Coral González-Martínez, Miriam Alcaide Lucena, Inés Cañas-García, Clara Bayarri, Juan Ramón Delgado, Encarna González, Jose Antonio Lorente, M. Jose Serrano

**Affiliations:** 1 GENYO Centre for Genomics and Oncological Research, formed by Pfizer, the University of Granada and the Andalusian Regional Government, PTS Granada, Liquid Biopsy and Cancer Interception Group, Av. de la Ilustración, 114, 18016 Granada, Spain; abel.garcia@genyo.es (A.G.-D.); maria.molina@genyo.es (M.P.M.-V.); coralgonma@correo.ugr.es (C.G.-M.); miriam.alcaide.lucena@gmail.com (M.A.L.); inescanasgarcia@gmail.com (I.C.-G.); ci.bayarri@gmail.com (C.B.); jose.lorente@genyo.es (J.A.L.); 2 Universidad Internacional de la Rioja, Avenida de la Paz, 137, 26006 Logroño, Spain; 3 Departamento de Medicina, Facultad de Medicina, Universidad de Granada, 18016 Granada, Spain; 4 Servicio de Cirugía General y del Aparato Digestivo, Hospital Clínico San Cecilio, 18016 Granada, Spain; 5 Department of Thoracic Surgery, Virgen de las Nieves University Hospital, Av. de las Fuerzas Armadas, 2, 18014 Granada, Spain; 6 Bio-Health Research Institute (Instituto de Investigación Biosanitaria ibs. GRANADA), Complejo Hospitalario Universitario Granada (CHUG), University of Granada, 18012 Granada, Spain; juanramondelgado@gmail.com (J.R.D.); encarnagonzalezflores@gmail.com (E.G.); 7 Laboratory of Genetic Identification, Department of Legal Medicine, University of Granada, Av. de la Investigación, 11, 18071 Granada, Spain; 8 Department of Pathological Anatomy, Faculty of Medicine, Campus de Ciencias de la Salud, University of Granada, 18016 Granada, Spain

**Keywords:** *TP53* mutations, liquid biopsy, cfDNA, CTC, tissue, concordance

## Abstract

**Simple Summary:**

Most solid tumors share mutations in *TP53* that is thus considered one of the main cancer driver genes. Mutations in *TP53* occur very early during tumor development, so their identification helps in diagnosing cancer. Furthermore, knowing in advance the *TP53* mutation status might help guiding targeted treatments against this gene. However, this analysis is mainly performed in tissue samples, that is, solid biopsies, being an invasive technique. Contrarily, liquid biopsies, consisting of the analysis of blood samples, are non-invasive, can be performed repeatedly, helping in monitoring the patient evolution, and might be useful in early stages when the tumor is not yet detected by other technologies. Here, we review the main studies conducted on two types of liquid biopsies: circulating tumor cells and cell-free DNA. We discuss the main findings regarding *TP53* mutation analysis, the clinical utility of this information and some controversies arising from the study of liquid biopsies compared to tissue samples, and we finish by suggesting future directions within this field.

**Abstract:**

Being minimally invasive and thus allowing repeated measures over time, liquid biopsies are taking over traditional solid biopsies in certain circumstances such as those for unreachable tumors, very early stages or treatment monitoring. However, regarding *TP53* mutation status analysis, liquid biopsies have not yet substituted tissue samples, mainly due to the lack of concordance between the two types of biopsies. This needs to be examined in a study-dependent manner, taking into account the particular type of liquid biopsy analyzed, that is, circulating tumor cells (CTCs) or cell-free DNA (cfDNA), its involvement in the tumor biology and evolution and, finally, the technology used to analyze each biopsy type. Here, we review the main studies analyzing *TP53* mutations in either CTCs or cfDNA in the three more prevalent solid tumors: breast, colon and lung cancers. We evaluate the correlation for mutation status between liquid biopsies and tumor tissue, suggesting possible sources of discrepancies, as well as evaluating the clinical utility of using liquid biopsies for the analysis of *TP53* mutation status and the future actions that need to be undertaken to make liquid biopsy analysis a reality for the evaluation of *TP53* mutations.

## 1. Introduction

The renowned tumor suppressor *TP53* is considered the guardian of the genome as mutations in this gene are found in virtually all solid tumors at different frequencies and facilitate adaptive responses to external stress conditions [1]. The effect of *TP53* mutations was broadly studied and it is well demonstrated that these mutations increase cancer risk and once cancer occurs, promote invasion, metastasis and chemoresistance, among other oncogenic mechanisms [2]. Furthermore, *TP53* mutations are more commonly found in patients with history of chemotherapy and/or radiotherapy [3], suggesting they might also arise as a consequence of treatment-derived stress. Finally, *TP53* mutations are also identified in healthy old individuals [4] arising from clonal hematopoiesis, implying that distinct mutations from distinct origins might contribute differently to cancer occurrence.

For decades, presence or absence of *TP53* mutations was mainly studied on tumor tissues, firstly using immunohistochemical techniques and finally involving next-generation sequencing technologies. However, there are well-accepted limitations of the use of solid biopsies, such as the invasiveness or the inability to study tumor heterogeneity and evolution that emphasize the need for a more comprehensive tool. The use of liquid biopsies as a diagnostic and prognostic tool is attracting interest due to its low invasiveness [5]. This facilitates the repetitive analysis of a particular biomarker to monitor the evolution of diseases like cancer, thus predicting their progression. The main types of liquid biopsies with a demonstrated prognostic role in several solid tumors are circulating tumor cells (CTCs) and cell-free DNA (cfDNA), particularly in advanced-stage tumors [6,7,8,9]. However, regarding diagnosis, and particularly at early stages, these biomarkers that need to be highly specific and sensitive have not yet proved their utility, although some studies suggest very low false positive rates [10,11].

Since *TP53* mutations are ubiquitously present in solid tumors, they are considered driver mutations originating cancer [1] but also can be studied as actionable, being thus targetable by drugs. Many different strategies are suggested to treat *TP53* mutant tumors such as restoring wild type *TP53*, inducing synthetic lethality, depleting the mutant protein or even affecting downstream targets [2]. There are several drugs targeting *TP53* mutant tumors, including adenoviral vectors that restore wild type protein [12], and they have shown their utility in several clinical trials (ClinicalTrials.gov Identifier: NCT02965950 or ClinicalTrials.gov Identifier: NCT03544723, among others). However, the mutation status is mostly analyzed through solid biopsies, limiting the potential use of these drugs.

The main difficulty in analyzing CTCs is their scarcity in blood (range of 1 to 50 in metastatic patients [13,14]), although improvements in the sensitivity of the isolation methods together with incorporation of high-resolution and throughput technologies, such as mass cytometry, are providing novel insights on the diagnostic role of CTCs. As CTCs are released from the primary tumor, they are expected to resemble somehow the tissue of origin and its molecular characteristics. However, CTC heterogeneity, mainly due to tumor evolution itself, makes CTC analysis even more complex [15]. Furthermore, CTCs need to escape the immune system to be able to metastasize and to do so, they might acquire novel mutations in the process [16] and can also interact with different immune cells such as platelets or neutrophils, enhancing their invasive potential [17]. Thus, CTCs are biological information containers showing the real status of the disease over time. Genetic evaluation studies on CTCs are dependent not only on the molecular technology used for analysis but also, and more importantly, on the isolation methodology for CTC enrichment. Most of the studies are performed using epithelial markers such as EpCAM for positive immunomagnetic selection [9,13,14,18], followed by single-cell isolation and sequencing techniques [16,19], whereas others use CTCs physical properties such as size filtration protocols for isolation of a wider range of CTC subpopulations [20,21] which might contribute to diverse and sometimes contradictory results.

Regarding cfDNA, although it is also present in blood at low concentrations (below 100 ng/mL in healthy individuals), its stability and the ease of its isolation and study compared with CTCs potentiates its use [22]. Different aspects of cfDNA are of importance: origin, concentration, integrity and genetic content. Regarding origin, cfDNA (cell-free DNA) must not be misinterpreted as ctDNA (cell-tumor DNA). Both are circulating nucleic acids, however, while ctDNA has exclusive tumor origin (demonstrated by mutation/genetic aberrations occurrence), cfDNA includes DNA from both the tumor and healthy tissues. It was described that cancer patients have increased levels of cfDNA in breast [23,24], colorectal [25] and lung [26] cancers, among others, having a prognostic role. This is supposedly related to higher cell turnover occurring in the tumor that will therefore release (actively or passively) ctDNA, generating greater amounts of cfDNA as a result. Furthermore, fragmentation of circulating DNA reveals not only the amount of time being exposed to DNases but also its epigenetic characteristics regarding transcriptional status (according to tissue origin) as these modifications might protect against degradation [27]. Thus, integrity levels vary depending on tumor type [28] and stage [29]. Finally, the genetic content of cfDNA is expected to picture the real status of the primary tumor from which it was released. Should this be true, identification of particular gene mutations/aberrations in cfDNA is of great importance for elucidating tumor heterogeneity as well as to predict treatment outcomes and thus serve as a prognostic tool.

Here, we will review the main studies on *TP53* mutation status on the most commonly studied liquid biopsies (CTCs and cfDNA) from the most prevalent tumors (colorectal, lung and breast cancers). We will be discussing the correlation with tissue mutation status as well as between the two liquid biopsies. Furthermore, we will present some data on the clinical utility of analyzing *TP53* mutations in liquid biopsies compared with tumor tissue genotyping and will finish considering some of the main concerns arising from their study in liquid biopsies.

## 2. Variability of TP53 Status between Liquid and Solid Biopsies

### 2.1. Circulating Tumor Cells (CTCs) and Tissue Correlation

Molecular evaluation of circulating tumor cells presents many challenges, among which the CTC isolation efficiency of each methodology may over-represent one CTC subpopulation over another [30]. Importantly the limit of detection (LoD) varies from 1 in 7.5 mL to 1 CTC in 1 mL of blood depending on the platform used and whether an enrichment step is performed before the analysis [31]. Furthermore, mutation analysis of single cells requires whole-genome amplification protocols that might potentially introduce technical errors [9,19,32], all of which might reduce mutation concordance with tissue (Table 1).

Presence of *TP53* mutations is an indicator of bad prognosis for breast cancer [33], thus triple negative breast cancer (TNBC), being the one with worse prognosis, is expected to harbor a great mutation frequency. In fact, according to the Cancer Genome Atlas Network, up to 80% of TNBC tissues present *TP53* mutations [34]. Regarding liquid biopsies, deleterious *TP53* mutations matching the tumor were identified together with wild type (WT) alleles in different CTCs from two TNBC patients, suggesting the presence of diverse CTCs populations [18]. However, these authors could not identify WT *TP53* in tumor tissue in contrast to WT CTCs, possibly due to its low frequency in the tissue compared with the mutant allele [18]. This heterogeneity was also described by Mu et al., 2016, who found heterozygous *TP53* mutant CTCs agreeing with the solid tumor mutation status but also homozygous CTCs for the mutation within the same patient [35], suggesting acquisition of a second hit during circulation. On the contrary, a recent study identified *TP53* mutations in CTCs from advanced breast cancer patients but not in tissue, suggesting that some of these mutations might be markers for metastasis in early disease [32]. Likewise, some *TP53* mutations identified in CTCs from either advanced disease [30] or metastasis [9] were absent in tissue possibly due to their low frequency. Furthermore, different genetic profiles (not only dependent on *TP53* but also other genes) were found in CTCs according to their phenotype, either mesenchymal (M) or epithelial (E). Regarding *TP53* mutations, the greatest proportion of mutations (25%) was found in M−/E− CTCs, followed by M−/E+ CTCs (23.1%) with a slightly lower mutation rate in M+/E+ CTCs (17.3%) and M+/E− (13.5%) [32].

As *TP53* is one of the main driver genes also in colorectal cancer (CRC), it is expected that mutations in this gene will be easily detected in CTCs from CRC patients. However, there is a lack of studies assessing the correlation between solid and liquid biopsies for this particular gene. In an early study, Khan et al., 2000 showed that *TP53* mutations were found in 46% (19/41) of their population of CRC patients, being invariably present at both the primary tumor and its matching liver metastasis. However, only a proportion of these mutations (42%; 8/19) was detected in CTCs [8]. They suggested high concordance between primary and metastatic sites, which might be due to the use of high-fidelity Taq compared to other studies, but they also pointed out their inability to detect low-allele frequency clones in CTCs, which might mask the heteroclonality of the tumor. Diversity regarding presence/absence of *TP53* mutations was identified in CTCs from colorectal cancer patients with mutation frequencies concordant with previous studies (38.7%; 12/31) but with some disparities with tissue showing wild type *TP53* [16].

Regarding lung cancer, the prognostic role of CTCs is well demonstrated [36], however their clinical utility is yet to be determined and regarding molecular evaluation, not many studies have assessed *TP53* status. Zhang et al., 2014 identified the same *TP53* mutations, previously detected in tissue, in cultured CTCs from the same patient [37], with a discordance rate of 44% suggesting genetic heterogeneity of both tissue and CTCs. Later, Pailler et al., 2019 identified the same mutation in CTCs and tissue, although at different variant allele frequencies (VAF), 100% and 53%, respectively [19], which might explain the difficulty in identifying particularly very low frequency alleles in CTCs. Recently, He et al., 2020 analyzed mutations in CTCs for a 50-gene panel (including *TP53*) and found consistencies between liquid and solid biopsies ranging from 43% to 93% [11].

Selective pressures within the microenvironment facilitate tumor cells evolution at both temporal and spatial scales. This creates a multifaceted portrait that sometimes cannot be pictured if only analyzing the more stable and static solid tissue. Thus, the main source of discrepancies between solid biopsies and CTCs is tumor heterogeneity that in fact is represented by different CTC subpopulations. Furthermore, single-cell mutation analysis tends to misrepresent the disease as a whole, being then more effective in capturing *TP53* mutations in advanced-stage tumors (with greater expected CTC numbers) than in early disease stages.

### 2.2. Circulating Cell-Free DNA (cfDNA) and Tissue Correlations

Concordance between mutations detected in cfDNA and solid biopsies has been assessed in many cancer types, achieving great correlations particularly in advanced tumors [38]. However, discordances might appear accounting for specific tumor types or stages [39] as well as for the methodology used for variant evaluation or even the time frame in which comparative samples were isolated [40]. In particular, when next-generation sequencing (NGS) technologies are used, the limit of detection (LoD) is around 0.1%, whereas the use of digital PCR (dPCR) or other more sensitive techniques offers an LoD as low as 0.001%, allowing detection of somatic as well as germline mutations even in healthy individuals with very low levels of cfDNA [41]. Importantly, as *TP53* mutation frequency is usually very high, methodology efficiency is expected to affect, to a lesser extent, its detection both in plasma and tissue (Table 1).

A meta-analysis was performed to evaluate the prognostic utility of cfDNA mutation status in breast cancer, showing a mutation rate for *TP53* of 37.8% independent of tumor stage [42], being much lower than those previously described in tissue. They demonstrated that *TP53* mutations detected in cfDNA were a good prognostic tool as they were associated with cancer recurrence, short disease-free survival and progression-free survival; however, there was a lack of diagnostic power particularly at early stages. Later, another meta-analysis was able to demonstrate the diagnostic utility of *TP53* mutations in cfDNA for advanced breast cancer with a diagnostic performance of 0.94 [43]. Frequencies of *TP53* mutation reported in these studies agree with some recent ones reporting frequencies of 34% in tissue [44,45] and of 31% [46] and 32.5% [47] in plasma of primary breast cancer patients. Nevertheless, mutation frequency is higher in some other studies, showing a *TP53* mutation prevalence of 41% [45], 50% [7] and 51.7% [24]. The fact that the reported variant allele fraction (VAF) of *TP53* mutations in plasma has such wide ranges may make detection difficult when at low VAFs and may make it overestimated when at high VAFs, being a source of discordances with tissue. Some authors found a VAF of *TP53* mutations ranging from 0.09% to 20.56% [46], from 0.07% to 50.3% [24] or from 2% to 70% [48]. Concordance rates for *TP53* mutations between breast cancer tissue and plasma are extremely different across studies, which might be dependent on disease subtype or stage [49] but also might be affected by time sampling bias, technology used for NGS analysis or the VAF for each *TP53* mutation presented in each patient. On the one hand, high concordant results were obtained by Shaw et al., 2017 who identified all (100% concordance) *TP53* mutations present in tissue in cfDNA [9]. Madic et al., 2015 also demonstrated 81% concordant results between the two types of biopsies [48]. Later, 50% discordances for *TP53* mutations were found by different authors [40], [50], and lower concordant results (36.4% and 22%) were reported by Chae et al., 2017 [51] and Rodriguez et al., 2019 [44], respectively. Finally, Delmonico et al., 2019 found no concordance for *TP53* mutations in paired cfDNA/tissue samples [46].

Prevalence of *TP53* mutations in cfDNA of colorectal cancer patients increases as the tumor progresses [52] and thus it is highly variable between studies depending on inclusion criteria. VAFs for *TP53* range from 80.9% [25], 74% [53] and 69.3% [54] to 34.2% [52] among different studies. Wang et al., 2004 identified *TP53* mutations in 36.5% (38/104) of colorectal cancer samples, of which 34.2% (13/38) were also identified in serum [52]. However, their serum samples included not only cfDNA but also genomic DNA from white blood cells as no specific cfDNA extraction kit was used. Furthermore, no mutations in *TP53* were found in cfDNA of any of the 26 patients without mutations in the tumor tissue, suggesting a good negative predictive value. More recently, greater correlation rates have been obtained between tissue and cfDNA, which might be due to improvement of the sensitivity of the methods or a more homogeneous patient selection. Mansukhani et al., 2018 developed a highly sensitive method for mutation detection in cfDNA and found that some *TP53* mutations were only present in cfDNA but not in tissue, although their overall concordance rate was 88%, accounting for the 12 genes analyzed [55]. Later, Cao et al., 2020 identified 81% concordance for *TP53*, being greater than that of other genes such as *APC* (67%) or *KRAS* (42%), which might confirm that *TP53* mutations occur earlier during tumor development [53] and are more easily studied in cfDNA.

For lung cancer, allelic frequencies of *TP53* mutations in cfDNA have very wide ranges, from 0.12% to 84.8% [29], suggesting they might either have originated early during the carcinogenic process (in patients with greater VAFs) or much later, even in disseminating CTCs (in patients with lower VAFs). Thus, the correlation between cfDNA and tissue samples for *TP53* mutations is expected to be variable. In another study, *TP53* mutations in cfDNA were only identified in 50% of the patients [47]; however, in another cohort of non-small cell lung cancer (NSCLC) patients, *TP53* mutations were found in 71.7% (81/113) of patients [7], which again might be due to different inclusion criteria. High concordance was found in 10 patients comparing *TP53* mutation status between tissue and cfDNA. Interestingly, VAFs were higher in plasma than in tissue (12.04% and 10.80%, respectively) [26], suggesting the origin of cfDNA might not only be tissue but also CTCs. In another study, *TP53* mutation frequency was 79% with greater frequency for advanced small-cell lung cancer (SCLC) than for localized SCLC (83% vs. 76%) [56]. Furthermore, these mutations were identified in cfDNA at earlier and later tumor stages even when tissue allele frequency was low (~20%). These data suggested they might be drivers for metastasis should the source of this cfDNA be disseminating CTCs [57]. The diagnostic sensitivity of cfDNA for lung cancer (including not only *TP53* mutations but also other cancer genes) was 68%, with 96% of the specificity and sensitivity rate increased (63%, 83% and 94%) according to tumor stage (for stages I, II and III, respectively) [58].

Detection of *TP53* mutations in cfDNA is highly dependent on the variant allele frequencies; likewise, CTCs are dependent on disease stage and tumor type. Thus, to better understand correlations between cfDNA and tissue mutation status, it is mandatory that the study population is completely homogeneous regarding disease stage. Furthermore, the impossibility of determining whether ctDNA originated from tissue or from disseminating CTCs might make interpretations of *TP53* mutation difficult, as discrepancies might be intrinsic to tumor evolution itself.

## 3. Correlation of *TP53* Status between CTCS and cfDNA

Analysis of circulating tumor cells as well as cfDNA is meant to represent the solid tumor from which they were released. However, each biomarker has some limitations, impeding 100% concordance between the two types of liquid biopsies as well as with the tissue of origin. For example, cfDNA is more capable of fully representing the tumor heterogeneity, whereas CTCs, depending on the isolation procedure used, are more limited in this aspect [5]. On the contrary, CTCs have the ability to provide proteomic, as well as RNA, information that is missed if only analyzing cfDNA, as well as the ability to picture the metastatic potential. Very few studies have assessed both CTCs and cfDNA within the same patient, and regarding *TP53* mutation status, results might be very dependent on tumor size, allele frequencies, sampling bias and even tumor evolution.

Number of CTCs (≥5/7.5 mL blood) in breast cancer was shown to be a bad prognostic marker in contrast with baseline (before treatment) ctDNA levels that, although detected more frequently than CTCs, did not have prognostic utility [48]. In this study, 70% of patients having a *TP53* mutation in either tumor tissue or plasma had at least one CTC detected and 52% more than five CTCs, although their number did not correlate with cfDNA levels. Conversely, Shaw et al., 2017 found a high correlation between total cfDNA levels and CTC counts (≥5), being both associated with poorer overall survival [9]. They were not able to detect a *TP53* mutation in either tissue or CTCs which, however, was detected at very low levels in cfDNA. Mutation analysis showed, in general, a good correlation for *TP53* between cfDNA and individual CTCs, although some mutations identified in tissue and cfDNA at very low frequencies could not be identified in CTCs possibly due to the scarcity of CTCs analyzed [9]. Beije et al., 2016 did not find any correlation between presence/absence of CTCs and either cfDNA levels or fragmentation status in colorectal cancer patients [59], which again might be due to the small sample size analyzed. Regarding lung cancer, a positive correlation was found between *TP53* VAF in plasma and CTC counts, being identified as significant predictors of shorter survival [56].

As the information provided by either CTCs or cfDNA reflects a different aspect of the disease, genetic data in both types of liquid biopsy should be analyzed as a complementary tool representing distinct cancer mechanisms. Rather than trying to assess the correlation between the two types, information on *TP53* mutation status might be used to illustrate tumor stage and evolution, identifying mutations that occurred either earlier or later depending on the VAF within each liquid biopsy.

## 4. Clinical Utility of *TP53* Mutation Identification in Liquid Biopsies

The lack of invasiveness characterizing liquid biopsies is an advantage that allows for monitoring patients over time and to evaluate tumor evolution and treatment responses. On the one hand, the prognostic utility of CTC numbers at baseline and even after treatment has long been demonstrated in several tumor types [59,60,61]; however, molecular analyses on CTCs as tissue surrogates might also give therapeutical information enabling precision medicine, and this has not yet been well studied. On the other hand, cfDNA levels as well as presence/absence of genetic aberrations also constitute a prognostic tool in breast [62], colorectal [63] and lung [64] cancers. Furthermore, monitoring variant allele frequencies (VAF) during treatment with identification of novel mutations as a consequence of a particular drug might provide insights into resistance mechanisms. Frenel et al., 2015 monitored for the first time VAF in cfDNA of 39 patients with different solid tumors (including breast, lung and colorectal) after treatment [65]. They were able to identify changes in the allelic frequencies of some genes (including *TP53*) that might be potentially used as tools to detect treatment resistance and recurrence/metastasis [52]. Since *TP53* mutations are widely present in most solid tumors, they are also associated with other driver mutations in actionable genes such as *BRCA1/2* for breast cancer [66,67] or *EGFR* for lung cancer [68], among others. Furthermore, coexisting mutations with *TP53* associated with treatment resistance allow stratifying patients for a particular treatment, thus enabling precision medicine, and are currently used in several clinical trials as a biomarker for treatment response (Table 2).

Regarding breast cancer treatment, presence of *TP53* mutations in cfDNA is associated with lower progression-free survival [50] independently of treatment, and increasing VAFs are associated with treatment resistance [69] and metastatic events [70]. Li et al., 2017 demonstrated that the mutant TP53 protein sensitizes to some biological therapies such as Lapatinib [71]. Furthermore, administration of PARP inhibitors such as Olaparib in patients with mutated *BRCA1/2* was shown to have beneficial effects, thus the association of *TP53* and *BRCA1/2* mutations might be used also as a therapeutical approach [67]. Finally, appearance of *TP53* mutations (or genes within the *PIK3CA/MTOR/PTEN* pathway) after anti-HER2 [72] or tamoxifen [73,74] therapy is an indicator of resistance and can be monitored using cfDNA.

With respect to colorectal cancer, many studies use cfDNA for monitoring patients’ response to therapy, particularly for metastatic ones. Regarding the VAF of *TP53* mutations, it was shown to decrease in patients that did not develop metastasis after primary colorectal cancer surgery, whereas its increase was associated with liver metastasis development [6]. Accounting for specific treatment regimes, a case study demonstrated that triple mutant patients (*APC/TP53/KRAS*) had complete remission after FOLFIRI + Bevacizumab treatment, suggesting that follow-up of these mutations by cfDNA should be performed [75]. As mutations in *TP53* were demonstrated to be a marker for *VEGF* expression, patients with these mutations are expected to benefit from the anti-VEGF treatment bevacizumab [76]. Co-occurrent mutations were also observed in CRC patients for whom *TP53* was identified mutated together with *KRAS.* In fact, Cao et al., 2020 found shorter progression-free survival in those without *KRAS/TP53* co-occurrent mutations [53]. Nevertheless, it is known that *TP53* mutations might also arise as consequence of a particular therapy, for example, in metastatic colorectal patients treated with Cetuximab; thus, its appearance may produce resistant clones that need to be monitored [77] and possibly targeted by different drugs.

The clinical utility of *TP53* mutations in lung cancer is still controversial as some authors showed that a decrease in their VAF after adjuvant chemotherapy was somehow related to better outcomes [78], whereas others found no correlation between *TP53* mutation status with chemotherapy response [79,80]. This might be due to the particular mutation detected, as some seem to be associated with sensitivity to treatment, whereas others are more likely resistant markers [79]. Regarding biological treatments, tyrosine kinase inhibitors (TKIs) are being successfully used to treat lung tumors with *ALK* rearrangement or when sensitizing *EGFR* mutations are present [80], as some mutations such as *EGFR* T790M are related to Gefitinib and Erlotinib resistance [21]. Furthermore, *TP53* mutations, either before or after treatment, are also associated with a limited response to TKIs [81]. Patients with baseline *TP53* mutations showed worse overall survival, and progression-free survival of patients having developed *TP53* mutations during treatment was comparable to those holding mutations prior to treatment [82]. An example of first-generation TKIs is Crizotinib, whose resistance has been related not only to *EGFR*-resistant mutations but also to occurrence of *TP53* mutations at both tissue [83] and liquid [19] biopsies. Second- and third-generation TKIs with distinct action mechanisms are being developed to overcome this resistance [84]. However, treatment resistance with Osimertinib, an example of a third-generation TKI, is also related to *TP53* mutations. In particular, Ding et al., 2019 identified four patients with novel *TP53* mutations in cfDNA after Osimertinib treatment with a VAF as high as 24% [85].

As previously described, the clinical utility of cfDNA mutation analysis is more widely demonstrated compared to CTC evaluation for prognosis and treatment response due to the ease of its study. Furthermore, the utility of CTC evaluation has become restricted to CTC number counts rather than molecular analysis. However, this fact does not undermine the clinical utility of CTCs. In fact, both CTC counts and cfDNA mutation analysis might be used in combination, together with imaging data as well as other clinical tools, to improve treatment monitoring

## 5. Discussion

Some of the mutations identified in plasma are shown to be derived from peripheral blood mononuclear cells (PBMCs) due to clonal hematopoiesis; however, the role or impact of these driver mutations in PBMCs of healthy donors is still controversial. Khan et al., 2000 did not find any *TP53* mutation in either healthy tissue or its matching peripheral blood of 10 healthy donors [8]. Likewise, no mutated *TP53* was detected in serum samples from 50 healthy volunteers [52] or single white blood cells from the two triple negative breast cancer patients analyzed as controls [18]. However, some gene mutations are identified in healthy elderly subjects that might be due to clonal hematopoiesis. In a study by He et al., 2016, six gene mutations in cfDNA were shared between healthy controls and NSCLC patients, however, mutations in *TP53* as well as in other genes were exclusively found in cancer patients [26], suggesting the mutation pattern may differ between cancer-affected and cancer-free individuals. Nevertheless, in another study, *TP53* mutations (most of which were missense-producing non-functional proteins) were identified in cfDNA of 11.4% (14/123) of healthy donors compared to 49% of small cell lung cancer (SCLC) patients and this result was further validated in an independent cohort of healthy donors (11/102) with similar (10.8%) mutation frequency [29]. Further, Schwaederle et al., 2016 identified *TP53* alterations in 1/222 (0.5%) healthy donors [47]. These were some of the first studies highlighting the challenges of using *TP53* mutations in cfDNA as a diagnostic marker since they demonstrated that cancer-free healthy individuals might hold mutations in this gene. Later, Hu et al., 2018 compared *TP53* mutation status in cfDNA, tissue and PBMCs from NSCLC patients and found that 42.4% (14/33) of variants were detected at both cfDNA and tissue, suggesting tumor-derived variants. However, they also identified 15.2% (5/33) of variants at both cfDNA and PBMCs but not tissue, suggesting clonal hematopoiesis [86]. In all of the above cases, the particular mutations detected were described according to HGVS recommendations, but no emphasis was made regarding mutation type (either loss-of-function or gain-of-function). If clinical implementation of *TP53* mutations detection in liquid biopsy is expected, characterization of these mutation types is mandatory, as different treatment regimens might be needed accordingly [87].

Even though *TP53* sequencing is considered the gold standard for mutation analysis in tissue, there is still debate over which protocol and technology are optimum [88]. Thus, regarding liquid biopsies, in which a greater sensitivity is needed and very low allele frequencies are expected, the difficulty in selecting the right protocol to produce reproducible results is even greater. The variety of platforms (mainly Illumina and Ion Torrent) and library preps (TruSeq, Ampli LowPass, AmpliSeq, KAPA HyperPlus, etc.), together with the development of specific cfDNA sequencing kits (Oncomine, Avenio, QIAseq, etc.), complicates the task. Thus, the lack of protocols standardization for the study of liquid biopsies keeps being a major challenge for their use as potent clinical biomarkers. Variability among results from different studies can be explained by many factors that need to be taken into consideration (pre-analytical and analytical phases as well as biological information intrinsic to the tumor itself) [45]. Further, regarding tissue analysis, FFPE might introduce some sequencing artifacts that need to be taken into consideration when analyzing discordances with liquid biopsies [89]. Furthermore, leaving aside tissue origin, disease stage and VAFs, the methodology used to assess *TP53* mutations significantly affects detection levels. Even though the correlation between dPCR and NGS methods was shown to be very high [90,91], some mutations were detected by one and not the other method. Of note, the region of study for a particular gene is also of great importance. Most early studies only analyzed *TP53* mutations in exons 4 and 8, comprising the majority of the known mutations [92] and later sequencing studies of the whole gene revealed lower concordances between liquid and solid biopsies, possibly due to the fact that rare mutations might be present at extremely low VAFs to be detected in both specimens [44]. In addition, genetic alterations such as methylation may also impact on *TP53* gene expression without the need for identifying point mutations [93], thus inclusion of further biomarkers in both solid and liquid biopsies might improve their diagnostic and prognostic utility. Moreover, the cost-effectiveness of each methodology needs to be considered if implementation into a clinical setting is expected. Regarding cfDNA analyses, their clinical use is only optimal when targeted approaches (such as direct *TP53* mutation detection) are used [5,66], compared to more comprehensive, genome-wide analyses. With respect to CTCs, the methodology used for isolation and subsequent analysis also has an impact on the cost–benefit balance of their application. Highly standardized FDA-approved technologies such as CellSearch have demonstrated their clinical utility [94], however there is still room for better phenotypic characterization and cost reduction; in fact, other platforms such as microfluidic-based ones are reducing reagents costs and improving performance [95] and might become more affordable in the near future.

All of this suggests that neither solid nor liquid are exclusive options but rather complementary tools [5,51]. In fact, the combination of several biomarkers/biospecimens has demonstrated to increase statistical power and to provide a clearer picture of the tumor heterogeneity. Wu et al., 2019 showed that the sensitivity of detecting *TP53* mutations in cfDNA was much improved when merging information from plasma, sputum and urine in lung cancer patients [96]. Interesting, however, was the fact that Cohen et al., 2018 had lower diagnostic performances (below 70%) in detecting colorectal, lung and breast cancers compared to other solid tumors combining cfDNA mutation analyses (including *TP53*) and proteomic information (CancerSeek) [97]. In their study, Cohen et al., 2018 developed a PCR-based assay to analyze cfDNA mutations in targeted driver genes with the intention of diagnosing cancer using the same 60 amplicons in many different tumor types. Their findings highlight the fact that particular tumor types (such as breast, colorectal and lung) might need specific biomarker patterns not necessarily consistent with other tumor types.

The heterogeneity of the tumor, which increases with tumor burden, disease evolution and treatment pressure may make identification of *TP53* mutations in different biospecimens (either solid or liquid biopsies) difficult. This genetic characterization is of great interest as it provides both diagnostic and prognostic information, as well as insights for targeted therapy and potential metastasis intervention. Application of liquid biopsies in combination with tissue samples will provide a broader picture of the tumor itself, with the benefit of analyzing *TP53* mutation status more comprehensively. However, there is still a lack of standardized protocols allowing full reproducibility among tumor types and laboratories, resulting in discordant and misinterpreted data. Further research needs to be performed on *TP53* mutation detection in liquid biopsies to provide reliable data able to complement solid biopsy information.

## Figures and Tables

**Table 1:** Frequencies of mutant and wild type (wt) *TP53* reported in different studies and calculated concordances between circulating tumor cells (CTC) and cell-free DNA (cfDNA) with tissue.

Cancer Type	Reference	CTC		cfDNA		Tissue		*N*	Concordance
		wt *TP53* Frequency	Mutant *TP53* Frequency	wt *TP53* Frequency	Mutant *TP53* Frequency	wt *TP53* Frequency	Mutant *TP53* Frequency		
Breast	18	0%	100%	N/A	N/A	100%	0%	2	100%
	34	3%	97%	N/A	N/A	3%	97%	30	100%
	31	53%	47%	N/A	N/A	82%	18%	17	0%
	30	33%	67%	N/A	N/A	17%	83%	6	83%
	9	100%	0%	60%	40%	80%	20%	5	100%
	42	N/A	N/A	30%	70%	0%	100%	10	40%
	43	N/A	N/A	62%	38%	66%	34%	32	84%
	44	N/A	N/A	86%	14%	83%	17%	58	0%
	46	N/A	N/A	15%	85%	0%	100%	26	88%
	39	N/A	N/A	20%	80%	30%	70%	20	70%
	48	N/A	N/A	61%	39%	35%	65%	23	65%
	49	N/A	N/A	76%	24%	64%	36%	45	76%
Colorectal	8	80%	20%	N/A	N/A	0%	100%	19	42%
	16	58%	42%	N/A	N/A	77%	23%	31	97%
	50	N/A	N/A	66%	34%	0%	100%	38	60%
	51	N/A	N/A	39%	61%	25%	75%	36	81%
	53	N/A	N/A	14%	86%	11%	89%	28	82%
	58	N/A	N/A	58%	42%	50%	50%	12	58%
Lung	36	60%	40%	N/A	N/A	47%	53%	15	73%
	19	0%	100%	N/A	N/A	33%	67%	3	67%
	26	N/A	N/A	90%	10%	50%	50%	10	60%
	56	N/A	N/A	76%	24%	63%	37%	120	68%

N, number of individuals studied; N/A, non-applicable refers to particular data not analyzed in a study.

**Table 2:** Clinical trials analyzing *TP53* mutations, among others, in liquid biopsies (either circulating tumor cells or cell-free DNA) as well as other biological samples.

Tumor Type	Stage	Liquid Biopsy	Other Samples	Methodology	Studied Mutations	Drugs	Phase	Participants	Identifier
Metastatic Breast Cancer	Unresectable locally advanced or metastatic HER2-positive	ctDNA	FFPE tumor tissue	NGS	*PIK3CA* and *AKT1* as well as in *TP53*, *ESR1*, *GATA3*, *ERBB2* and *PTEN*, amongst others	Ipatasertib, Trastuzumab, Pertuzumab	1	25	NCT04253561
Colorectal Cancer	I, II and III	ctDNA	FFPE tumor tissue	targeted resequencing and ddPCR	*KRAS, NRAS, BRAF, PIK3CA, TP53* and *APC*	N/A	N/A	1000	NCT04050345
Metastatic NSCLC	Advanced biopsy-proven metastatic NSCLC	cfDNA	Tumor biopsy	IHC and NGS	*EGFR* with concurrent *RB1* and *TP53* alterations	Osimertinib, Platinum (Cisplatin or Carboplatin) and Etoposide	1	30	NCT03567642
LUSC and HNSCC	Metastatic SCC of the lung or head and neck	cfDNA and gDNA	Tumor biopsy	Sanger sequencing and ddPCR	*MET* and *TP53*	N/A	N/A	80	NCT03938012
NSCLC	I–IIIA	CTCs and ctDNA	Tumor biopsy	NGS and ddPCR	*AKT1, KRAS, NRAS, BRAF, DDR2, EGFR, FGFR1, ERBB2 (HER2), MEK1, MET, PIK3CA, PTEN, TP53, MDM2, SOX2* and *P63*	N/A	N/A	50	NCT03771404
NSCLC	IIIB–MIV	CTCs	N/A	N/A	*PD-L1, MSI-H/dMMR, TMB, HLA, POLE, POLD1, DDR, TP53, KRAS, BRCA2, PBRM1, MDM2/4, EGFR, ALK, PTEN, JAK1/2, DNMT3A* and *STK11*.	Elemene plus first-generation EGFR-TKIs/first-generation EGFR-TKIs	4	468	NCT04401059
NSCLC	III not suitable for curative treatment or IV	ctDNA	Tumor biopsy	NGS	*ALK* fusion and *TP53* alterations	Brigatinib/TKI	2	116	NCT04318938

Abbreviations: ctDNA, circulating tumor DNA; gDNA, genomic DNA; CTC, circulating tumor cell; NGS, next generation sequencing; IHC, immunohistochemistry; ddPCR, digital droplet PCR; N/A, non applicable.
